# Supplementary material for: Evidence-Based Application of Acupuncture for Pain Management in Companion Animal Medicine
Source: Vet Sci. 2022 May 26;9(6):252. doi: 10.3390/vetsci9060252 (PMC9227989; doi:10.3390/vetsci9060252)
Supplement: Supplementary file 1 [file vetsci-09-00252-s001.zip › vetsci-1683605-Supplementary Materials.pdf]

veterinary sciences

MDPI

Table S1. Commonly used Pain points (Data adapted from Ref. [4]).

Pain State and Location	Common Point Used
**General Pain**	LIV-3, GB-34, Bl-60, GV-20, SP-6
**Inflammation**	LI-4, GV-14, LI-11
**Neuropathic Pain**	ST-36, PC-6, TH-5
**Neck Pain**	Jing Jia Ji, SI-3, BL-23, BL-24, BL-25
**Hip Pain**	GB-29, GB-30, BL-54, BL-60
**Elbow pain**	SI-8, PC-3, HT-1, LI-11
**Back Pain**	BL-40, *Shen Shu, Bai- Hui, Hua-tuo Ja Ji*
